# Detection and Classification of Human Body Odor Using an Electronic Nose

**DOI:** 10.3390/s90907234

**Published:** 2009-09-09

**Authors:** Chatchawal Wongchoosuk, Mario Lutz, Teerakiat Kerdcharoen

**Affiliations:** 1 Department of Physics and Center of Nanoscience and Nanotechnology, Faculty of Science, Mahidol University, Ratchathewee, Bangkok 10400, Thailand; E-Mail: g5037004@student.mahidol.ac.th; 2 Materials Science and Engineering Programme, Faculty of Science, Mahidol University, Ratchathewee, Bangkok 10400, Thailand; E-Mail: mario_lutz@web.de; 3 NANOTEC Center of Excellence at Mahidol University, National Nanotechnology Center, Bangkok 10400, Thailand

**Keywords:** E-nose, body odor, biometrics, PCA, deodorant, humidity correction algorithm

## Abstract

An electronic nose (E-nose) has been designed and equipped with software that can detect and classify human armpit body odor. An array of metal oxide sensors was used for detecting volatile organic compounds. The measurement circuit employs a voltage divider resistor to measure the sensitivity of each sensor. This E-nose was controlled by in-house developed software through a portable USB data acquisition card with a principle component analysis (PCA) algorithm implemented for pattern recognition and classification. Because gas sensor sensitivity in the detection of armpit odor samples is affected by humidity, we propose a new method and algorithms combining hardware/software for the correction of the humidity noise. After the humidity correction, the E-nose showed the capability of detecting human body odor and distinguishing the body odors from two persons in a relative manner. The E-nose is still able to recognize people, even after application of deodorant. In conclusion, this is the first report of the application of an E-nose for armpit odor recognition.

## 1. Introduction

Nowadays, electronic noses (E-nose) are well-known as efficient analytic devices that are widely used for many applications such as quality control of foods [1–5] and beverages [6–9], public safety [10,11], air protection [12,13] and medical applications [14–18]. Recently, there have been increasing interests in the application of E-nose for measurement of human body odors. If successful, many new applications await in such area as healthcare monitoring, biometrics and cosmetics. In principles, the human body dynamically generates unique patterns of volatile organic compounds (VOCs) under diverse living conditions such as eating, drinking, sexual activities, health or hormonal status [19]. These VOCs released from the human body can give some information about diseases, behavior, emotional state and health status of a person [20]. In addition, body odor is one of the physical characteristics of a human that can be used to identify people [21]. The human odor is released from various parts of body and exists in various forms such as exhalation, armpits, urine, stools, farts or feet. Natale *et al*. [22] developed an E-nose that can diagnose the urine odor of the patients with kidney disorders. Phillips and co-workers demonstrated the detection of lung cancer [23] and breast cancer [24] from human breath using E-nose. An E-nose was also tested to help monitor alcoholic consumption of aged persons by measuring the odors from exhalation [25]. However, to our best knowledge, no report is yet available on E-nose monitoring of human armpit odor. In fact, the armpit is a skin region where a vast number of glands and bacteria cooperate to produce a strong smell [26,27]. It can be the best source for sampling volatile chemicals released from the human body, which may give a unique pattern allowing identification of different persons.

One important obstacle to the detection the human body odor from armpits is sweat. Each day, humans produce different quantities of sweat, depending on the environment and, more importantly, life activities. Since it is well-known that most gas sensors are to some extent sensitive to humidity [28] this varying sweat content can be a problem for measurement of armpit odor samples. Therefore, a correction of the humidity effect is necessary to ensure a pure sensor response to only the volatile organic compounds that match with the identity of individual persons. Another problem for E-nose measurement of armpit odor is the disturbance from artificial chemicals such as deodorants because most adult people utilize deodorants to reduce unpleasant body odor. An interesting question arises whether E-nose can identify persons using deodorant or not? If both problems can be solved, biometrics based on armpit odor recognition would become viable.

In this paper, we propose a strategy to identify persons based on measurement of human body odor from armpits. To demonstrate this concept, we have designed and constructed an E-nose based on a set of metal oxide gas sensors. With this E-nose and the proposed method, identification of two persons either with or without using deodorant could be achieved.

## 2. Experimental

### 2.1. E-Nose System

**Figure 1.**
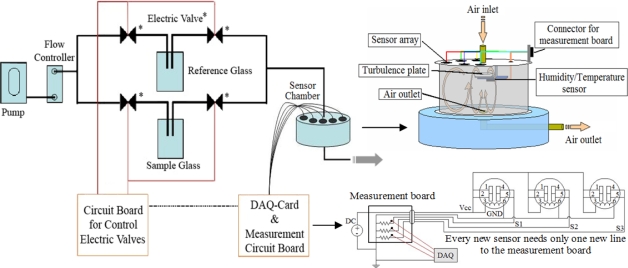
Schematic diagram of the lab-made E-nose system.

Our lab-made electronic nose system (see Figure 1) was designed to measure VOCs generated from the human body. It is comprised of three main parts: (i) sensor chamber (ii) air flow system and (iii) data acquisition (DAQ) and measurement circuit.

In this work, we have used gas sensors as commercially available from Figaro Engineering Inc. The gas sensors, as listed in Table 1, were selected in order to cover the targeted gases present in human body odor [29]. These gas sensors have been widely known as TGS (Tagushi) gas sensors since they were invented and patented by Naoyoshi Tagushi. The TGS gas sensors are usually produced by deposition of a metal oxide semiconductor (MOX), i.e., SnO_2_ and WO_3_, as a thin film on interdigit electrodes. Upon catalytic reactions of the metal oxide surface with the target gas molecules, usually at a temperature between 250–350 °C, the resistance between the electrodes is changed and measured. This type of gas sensors (resistive) has an advantage over other types of gas sensors (i.e., gravimetric or capacitive) that a simple circuit is required for implementation. Illustration of the circuit diagram and other details of each sensor can be obtained from the manufacturer website at http://www.figaro.co.jp/. The temperature and humidity sensor (SHT15; SENSIRION Inc.) was installed inside the sensor chamber. The sensor chamber is made of a glass cylinder sealed with Teflon plates on top and bottom. Both Teflon plates have an inlet and exhaust hole aligning oppositely. Under the inlet hole, there is a small Teflon plate to obstruct the stream of flow-in air, in order to create a turbulent that will assist in sensing by the gas sensors placed underneath the top cover. The temperature and humidity sensors are also mounted underneath this barrier plate. The air flow system consists of four electrically controlled solenoid valves, sample and reference glass containers, plastic pipes, and mass flow controller. Noteworthy, it is necessary for this type of measurement to switch between the reference and the sample glass containers. Four electrically controlled solenoid valves were used to avoid mixing of the gas from the reference and the sample. The gas either from the reference or sample containers was set to flow into the sensor chamber at a flow rate of 150 ml/min. For the measurement circuit, data acquisition was realized by a USB DAQ device (NI USB-6008) from National Instruments where each sensor voltage was measured with a dedicated channel of the DAQ device. The measurement software was written under LabVIEW package. The voltage divider method was employed for measuring the resistance of each sensor. The DAQ device was configured to acquire 2,000 samples at a time with a sampling rate of 2,000 samples per second for each channel. This generates an array of data that spans one second. To obtain noise reduction and higher precision, these 2,000 samples were then averaged to obtain only one value per second per channel. The resulting values were then recorded in a file for subsequent analyses.

**Figure 2.**
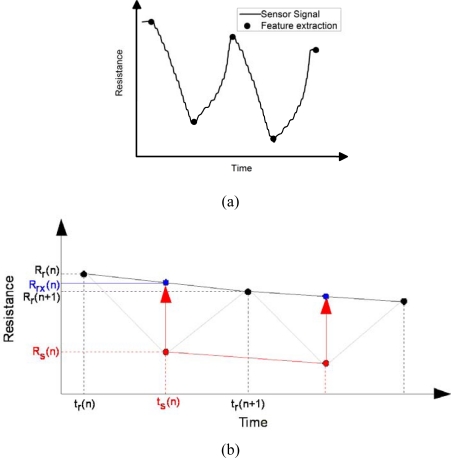
(a) Typical raw data from a sensor and the max/min feature extraction on each curve. (b) Correction method of baseline shift as time proceeds.

In our study, only simple features, i.e., the maximum and minimum resistances as obtained from switching between the reference and sample, were extracted and used for analyses, as shown in Figure 2a. The maximum and minimum resistances were the averaged values of their 10 neighboring data points. Since there is a gradual change in the reference and sample resistances over time, it is necessary to correct such baseline shift as time proceeds (See Figure 2b).

From Figure 2, index r denotes the reference, while index s denotes the sample. Index rx is defined as a baseline-corrected reference value. The variable (n) represents the running number of measurement loop (switching between the reference and sample). A linear interpolation is used as a baseline connecting between two reference points [*R_r_*(*n*) and *R_r_*(*n* + 1)]. The corrected reference point [*R_rx_*(*n*)] is calculated by projecting the sample point onto the baseline. As a result, the baseline-corrected difference between the sample and reference resistances is calculated via the following formula:

(1) $$\Delta R(n)=R_{s}\;(n)-R_{\mathrm{rx}}\;(n)$$

(2) $$\text{Where}\;R_{\mathrm{rx}}\;(n)=\frac{R_{r}\;(n+1)-R_{r}\;(n)}{2}+R_{r}\;(n)$$

For later data analysis and to compare data of different types of sensors, it is better to calculate the percentage change of resistance:

(3) $$\%R(n)=\frac{R_{s}\;(n)-R_{\mathrm{rx}}\;(n)}{R_{\mathrm{rx}}\;(n)}\times 100$$

### 2.2. Humidity Control

**Figure 3.**
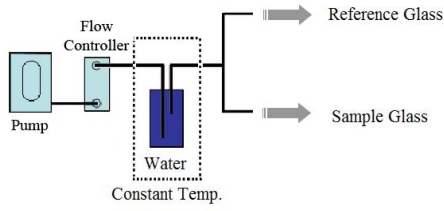
Schematic diagram of humidity control using hardware-based method.

As discussed in the introduction, most chemical gas sensors are sensitive to humidity. Therefore, if two identical samples with a different humidity are measured, the results can be different. In our work, we propose two methods as solutions to this problem. The first is a hardware-based method, where the sample was handled so as to have almost the same humidity as the background. Under such condition, the humidity signals will be equivalent for the sample and the reference, thereby only signals from the odors of interest result. To produce a constant humidity background, the carrier gas was directed to flow through a liquid water container that is immersed in a temperature-controlled heat bath (see Figure 3). The temperature of the heat bath can be adjusted until the generated humidity reaches the desired value. It is intuitive to anticipate that the native humidity would be lower than the higher generated humidity. We have done an experiment to investigate whether the generated humidity could overcome the native humidity of the samples. A humidity sensor was installed inside the sensor chamber. The temperature of the heat bath was adjusted until the reference humidity reached a desired value of 25%, 50% and 75%, respectively. Then, the humidity difference between the reference and the sample was compared and discussed (Section 3.1).

The second solution to the humidity problem is a software-based approach. A mathematical model describing the resistance of each gas sensor at different humidity level can be calibrated to subtract the humidity signal from the total signal. Although each aforementioned approach can be used independently, we have employed both schemes concurrently to achieve maximum accuracy. In addition, it should be noted that these humidity corrections could be applied in other E-nose systems or in the field conditions. Samples other than the human body odor can also be used with this algorithm. However, varying humidity (10–90%) should be tested before a measurement of a desired sample.

### 2.3. Human Body Odor Collection

Human body odors from armpits were collected from two male volunteers. The experiment was performed for five days with a sample collection of the armpit odors in the morning right after waking up (the volunteers typically wake up around 7–8 am) and in the afternoon (8 hours later). Cotton pads were used to transfer the odors from the armpits to the E-nose. A cotton pad must be in direct contact with the armpit for 10 minutes and stored in a special sample glass bottles with a screw-on closure. Once the morning samples were collected, the glass bottles were transferred to laboratory for E-nose measurement. To minimize the odor change due to bacteria, the samples, transferred via a heat-protection container, were measured within 30–50 minutes after sample collection. For the afternoon samples, E-nose measurement can be done immediately after odor sampling.

During the experiment period, the volunteers were requested to go about their ordinary life and activities: for example, they took a shower twice a day (before going to bed and after waking up following the morning sample collection). To avoid fluctuation in odor samples, they were not allowed to have sex and/or consume alcohol. To study the effects from deodorant, the volunteers were requested to use deodorant, after taking shower in the morning, but only on the right arm.

### 2.4. Evaluation of Sensor Response to Body Odor Strength

There are more refined and less subjective ways to measure odor strength in direct way. For instance, the concept of dilution-to-threshold principle can be used quite accurately to reduce uncertainties associated with subjective impressions [30–32]. In the cosmetic industry, human olfaction has been commonly employed to evaluate the odor strength of armpit for the development of deodorants. The armpit odor comprises a complex set of chemicals. Previously, isovaleric acid and volatile steroids (such as androstenone, androstadienone and androstenol) were thought to be the major contributors to armpit odor. However, armpit odor having more distinct and pungent oder involves the presence of other volatile compounds as well [33–35]. To simplify the odor strength of armpit, only a single component such as isovaleric acid can be used for training the sensory panel [36] and representing the sweaty primary odor [37,38] that contributes mainly to the armpit malodor. Hooper *et al* [36,39] assigned the concentrations of isovaleric acid levels on a scale 0 to 5 corresponding to subjective impression by using human nose, as shown in Table 2. Their test was carried out by a team of three female assessors of ages ranging from 20 to 40 years. They were selected for olfactory evaluation on the basis that each person is able to rank correctly the odor levels of the series of aqueous isovaleric acid solution listed in Table 2. The scale 0 to 5 has been usually used to represent the intensity of the armpit smell in the cosmetic industry. The judges are trained to memorize this scale and classify the odor strength of the samples. In this work, we evaluated the performance of E-nose in classification of body odor strength using isovaleric acid solutions prepared according to the intensity scale. A cosmetic face-cleaning pad with 0.15 mL of aqueous isovaleric solution was placed into a glass container for measurement.

## 3. Results and Discussion

### 3.1. Humidity Control

**Figure 4.**
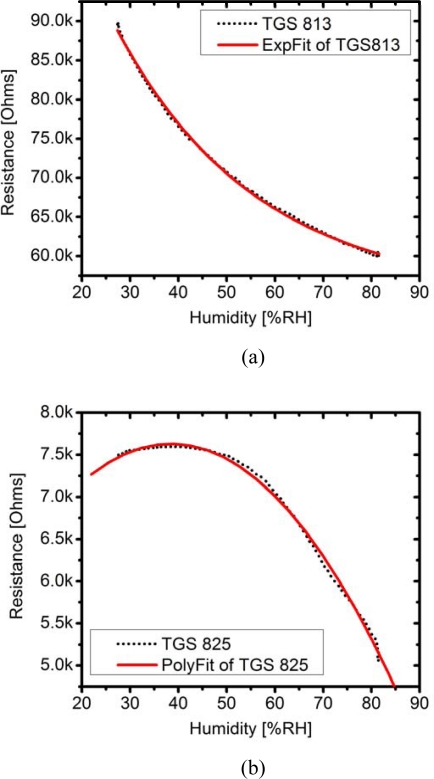

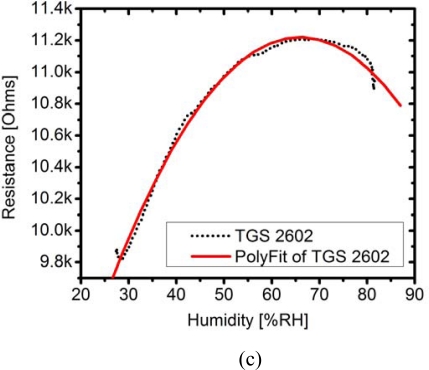
Resistance of sensors (a) TGS 813, (b) TGS 825 and (c) TGS 2602 versus relative humidity.

To investigate the sensor response to humidity, the relative humidity [%RH] was varied from 30% to 80%. Resistances arisen from humidity of TGS 813, TGS 825, and TGS 2602 sensors are displayed in Figure 4a,b,c, respectively. The graphs for the behaviors of TGS 822 and TSG880 (not shown in this paper) are similar to TGS 813. Mathematical models for the sensors’ response to humidity can be fitted via the following formulations:

Exponential equation of TGS 813:

(4) $$Rs_{813}=86682.00\text{exp}\left(\frac{-[\%\mathrm{RH}]}{29.05}\right)+55063.48$$

Exponential equation of TGS 822:

(5) $$Rs_{822}=24931.58\text{exp}\left(\frac{-[\%\mathrm{RH}]}{37.48}\right)+9054.41$$

Exponential equation of TGS 880:

(6) $$Rs_{880}=90496.88\text{exp}\left(\frac{-[\%\mathrm{RH}]}{36.33}\right)+55135.22$$

Polynomial equation of TGS 2602:

(7) $$Rs_{2602}=6958.22+129.172[\%\mathrm{RH}]-0.9788{\left[\%\mathrm{RH}\right]}^{2}$$

Polynomial equation of TGS 825:

(8) $$Rs_{825}=5646.63+103.26[\%\mathrm{RH}]-1.34{\left[\%\mathrm{RH}\right]}^{2}$$

These mathematical models were included in the data acquisition and analysis codes, thus allowing the response of sensors to humidity of the samples to be corrected on the fly. However, to achieve the maximum accuracy, hardware correction as shown in Figure 3 is co-employed. It was expected that, if the generated background humidity dominates the humidity of the sample, the effect arising from the humidity difference between the reference and the sample would be minimized. We have tested this assumption by measuring the armpit odor sample of a volunteer. Various humidity references, e.g., 25%, 50% and 70%, were generated and flowed through the sample. Table 3 shows absolute average percentage changes of resistances of each sensor and their standard deviations (or standard error) upon varying humidity background. Each average percentage changes of resistance presented in Table 3 was obtained by averaging data from three repeated measurements, in which each measurement performs switching between the reference and sample for five cycles (in total 15 datasets of each sensor were averaged). It was found that the change of the resistance between the reference (pure cotton pad) and the sample (cotton pad + sweat + armpit odor) and its error value become smaller when the background humidity was increased from 25% to 75%. The variation in each measurement, as implied by the standard error, was also reduced from ±51% with low humidity background to ±7% with higher humidity background, indicating that fluctuation in the dynamic measurement was also reduced. The decreasing error indicates less fluctuation of the sensor response arising from the humidity in sweat. At a lower relative humidity, the sweat in the cotton pad can evaporate much easier and contributes in a large part for the difference of resistances between the reference and the sample. At a higher relative humidity, the generated humidity weighs off the native humidity of the sample, thereby reducing the humidity difference between the reference and the sample. As shown on the right-most column of Table 3, the humidity difference between the reference and the sample decrease from 2.8% to only 0.29% when the generated humidity at 25% was replaced by higher relative humidity at 75%. However, saturated humidity (100%) is not recommended because it will suppress evaporation of odor molecules, which deteriorates the measurement.

The hardware-based method helps to reduce the effect arising from the humidity reference between the reference and the sample. It can be said that the sensor signals consist in a large part of the contribution from the odors of interest. Thus, relative humidity at 75% was applied in all experiments for measuring the human body odors in this paper.

### 3.2. Evaluation of Sensor Response to Body Odor Strength

**Figure 5.**
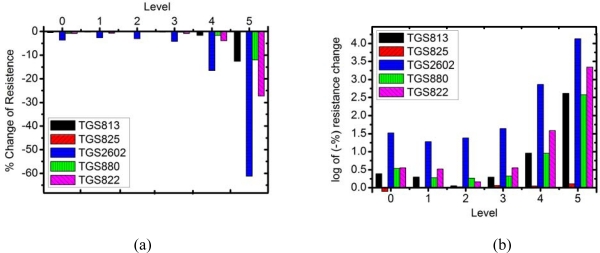
(a) Sensor response to isovaleric acid at different intensity level. (b) Logarithmic plot of the sensor response.

The response of each sensor to the isovaleric acid prepared according the intensity levels 1 to 5 is displayed in Figure 5. It can be seen that all sensors can discriminate intensity level 3, 4 and 5, but fail to distinguish between levels 0, 1 and 2. TGS2602 exhibits the highest response to the isovaleric acid. Since the intensity level of isovaleric acid has an exponential relation with the concentration (as seen in Table 2), the sensor response may be mathematically adjusted in order to understand the relationship between the sensor response with the intensity level, using the logarithmic function:

(9) $$Y=\text{ln}\left(1-\%R_{S}\right)$$

As shown Figure 5b, the logarithm of sensor response is linear with the odor strength for the intensity levels 3–5. The intensity threshold to isovaleric acid for all sensors is the intensity level 3. It implies that our E-nose may be limited to classify odor strength of persons who have at least definite armpit smell. Therefore, we have chosen two volunteers who have moderate armpit odors for E-nose measurement.

### 3.3. Detection and Classification of Human Body Odor

**Figure 6.**
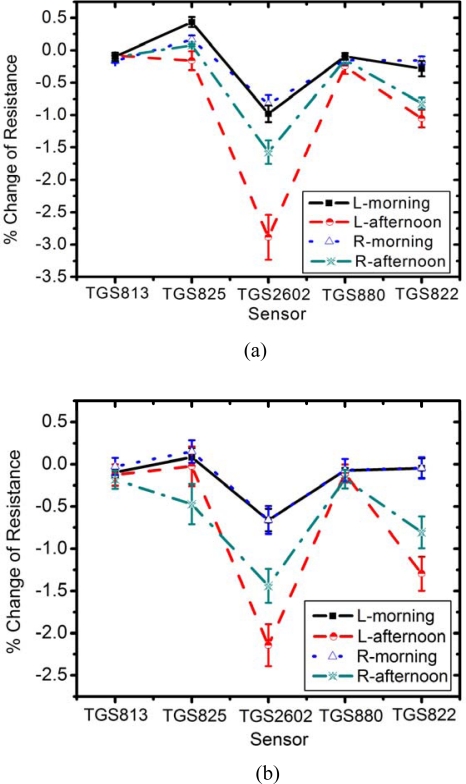
The sensor response with error bar of (a) person A and (b) person B in the morning and the afternoon. L and R denote the left and right armpits, respectively.

Armpit odors of two volunteer persons were measured by an E-nose during five days using a combined hardware/software humidity correction. Figure 6a,b exhibits the average of sensor response over 5 days as measured on the left (denoted by L) and right (denoted by R) armpits.

From Figure 6, it was found that TGS822 and TGS2602 have a high response to human body odor, in agreement with previous tests with isovaleric acid. Odor analysis of each person gives an interesting pattern. In the morning, both volunteers have only weak armpit odor, while the odor strength increases markedly in the afternoon. It can be seen from all sensors that the left and right arms of a person yield almost equivalent signals in the morning, since no deodorant was allowed before sample collection. In contrast, the afternoon results of the left and right arms deviate distinctly. The deodorant-free left armpit expresses noticeably higher signal strength. The difference of signal strength between morning and afternoon of each armpit was tested using a paired t-test with a significance level of 95% confidence (P = 0.05). Both person A and person B have a similar pattern in the difference of signal strength between morning and afternoon. For the person A, the mean difference of left armpit and right armpit between morning and afternoon are 1.500% change of resistance (P = 0.036) and 0.700% change of resistance (P = 0.250), respectively. In case of person B, the mean difference of left armpit and right armpit between morning and afternoon are 1.365% change of resistance (P = 0.004) and 0.775% change of resistance (P = 0.054), respectively. The paired t-test shows that the signal strength of deodorant-free left armpit (P < 0.05) have statistically significant difference between morning and afternoon at the level of 95% confidence. In contrast, the changes of deodorant right armpit between morning and afternoon (P > 0.05) did not reach the level of statistical significance. In general, deodorants suppress the armpit smell by reducing bacterial activity. Hence, an interesting question arises, “can deodorant blind human identification by an E-nose ?”

To allow an identification of human odors from two persons, we have adopted a pattern analysis based on principle component analysis (PCA). Only the data from the afternoon measurement was introduced into PCA. PCA is a popular statistical technique usually used to visualize in two or three uncorrelated dimensions transformed from all correlated information. In principles, PCA process contains five following steps:

1. Get data from matrix, X*_M×N_*. The row M represents different repetition of the experiment and the column N represents the number of independent sensors. In our case, M equals to 20 and N equals to 5.
2. Normalize the data matrix, *Norm*(X*_M×N_*), by the mean subtraction. The mean of each N column is calculated and subtracted from the data set. Hence, the new data set has a zero value of mean.
3. Calculate the covariance matrix, *Cov*(X*_M×N_*), and calculate eigenvectors and eigenvalues of the covariance matrix. The calculated eigenvectors must be unit eigenvectors.
4. Rearrange the eigenvectors and eigenvalues. The eigenvectors are ordered by eigenvalues from highest to lowest, ${\vec{\left(\mathrm{Cov}\left(X_{M\times N}\right)\right)}}_{\text{max}\to \text{min}}$.
5. Obtain the PCA result by matrix multiplication and transpose, ${\left({\vec{\left(\mathrm{Cov}\left(X_{M\times N}\right)\right)}}_{\text{max}\to \text{min}}⊗\mathrm{Norm}\left(X_{M\times N}\right)\right)}^{T}$. The obtained new dataset with orthogonal linear transformation are usually plotted in two or three dimensions containing the most relevant of the data set.

**Figure 7.**
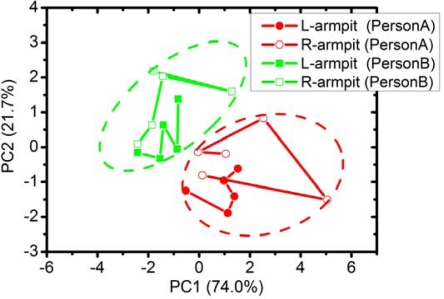
The 2D-PCA of armpit odors from two persons as measured in the afternoon during 5 days.

The PCA result is shown in Figure 7. The first principal component (PC1) explains 74.0% of the total variance and the second principal component (PC2) contributes 21.7% of the variation. The PCA result obviously distinguishes person A from person B. It indicates that each person has a specific odor pattern, even though these people have a similar life style. After both persons arrived at the laboratory, they have spent most of the time under the same humidity and temperature. The afternoon sample collection took place almost at the same time and the samples were subjected to measurement immediately. Therefore, the afternoon measurement should be more reliable than the morning one in which the odor change from bacteria could occur. The use of deodorant may not change the odor fingerprint, though it undoubtedly reducing the strength of a key chemical such as isovaleric acid that exerts strongly on perception of body’s smell. In Figure 7, each data point is plotted from day 1 to day 5. It can be seen that the odor of both armpits change everyday but PCA can still group the data of each person together. Therefore, E-nose can be a prospective candidate for identification or authentication of a person like other biometrical technologies [40].

## 4. Conclusions

In this paper, the detection and classification of human body odor by E-nose measurement have been demonstrated. We have proposed a scheme to minimize the humidity effect that is usually a serious problem for the detection of human body odor. This scheme employs a humidity generator (hardware) for creating smooth background humidity that can dominate the humidity of samples, as well as mathematical models (software) for humidity response of all sensors that can be used to eliminate signals from the humidity in a real-time fashion. The E-nose in conjunction with PCA method was shown to differentiate the body odors of two persons with similar life style and activities. In addition, we have found that deodorant does not effect the relative identification of these two persons. In order to extend the discrimination of human body odors beyond two persons, a number of improvements are required such as increasing sensor types that response to a variety of volatile molecules. It is hoped that the preliminary results presented in this paper will open the door to the field of human body odor biometrics.

## Figures and Tables

**Table 1.** Specifications of each metal oxide sensor.

**Sensor**	**Target Gas**	**Typical Detection Ranges**	**Heater Power Consumption**
TGS 813	Combustible gases	500–10,000 ppm	835 mW
TGS 822	Organic solvent vapors	50–5,000 ppm	660 mW
TGS 825	Hydrogen sulfide	5–100 ppm	660 mW
TGS 880	Cooking vapors	10–1,000 ppm	835 mW
TGS 2602	Air contaminants	1–30 ppm	280 mW

**Table 2.** The concentration of the isovaleric acid levels that correspond to subjective impression by using human nose.

**Level**	**Concentration of aqueous isovaleric acid solution (mM)**	**Subjective impression**
0	0	No odor
1	0.12	Slight
2	0.48	Definite
3	1.99	Moderate
4	7.88	Strong
5	32.33	Very strong

**Table 3.** The absolute average percentage change of resistance of each sensor upon varying humidity generated by hardware correction.

**Background humidity**	**TGS813**	**TGS825**	**TGS2602**	**TGS880**	**TGS822**	**Humidity sensor**
25%	3.948 (±55%)	2.211 (±38%)	3.727 (±38%)	4.765 (±37%)	5.529 (±43%)	2.823 (±51%)
50%	0.526 (±16%)	0.104 (±23%)	0.264 (±27%)	0.702 (±25%)	2.150 (±25%)	0.550 (±20%)
75%	0.158 (±4%)	0.057 (±8%)	0.581 (±4%)	0.160 (±5%)	0.185 (±7%)	0.293 (±7%)
